# Transcriptional changes in litchi (*Litchi chinensis* Sonn.) inflorescences treated with uniconazole

**DOI:** 10.1371/journal.pone.0176053

**Published:** 2017-04-18

**Authors:** Yongzan Wei, Chen Dong, Hongna Zhang, Xuewen Zheng, Bo Shu, Shengyou Shi, Weicai Li

**Affiliations:** Key Laboratory of Tropical Fruit Biology (Ministry of Agriculture), South Subtropical Crops Research Institute, Chinese Academy of Tropical Agricultural Sciences, Zhanjiang, China; The National Orchid Conservation Center of China; The Orchid Conservation & Research Center of Shenzhen, CHINA

## Abstract

In *Arabidopsis*, treating shoots with uniconazole can result in enhanced primary root elongation and bolting delay. Uniconazole spraying has become an important cultivation technique in controlling the flowering and improving the fruit-setting of litchi. However, the mechanism by which uniconazole regulates the complicated developmental processes in litchi remains unclear. This study aimed to determine which signal pathways and genes drive the responses of litchi inflorescences to uniconazole treatment. We monitored the transcriptional activity in inflorescences after uniconazole treatment by Illumina sequencing technology. The global expression profiles of uniconazole-treated litchi inflorescences were compared with those of the control, and 4051 differentially expressed genes were isolated. KEGG pathway enrichment analysis indicated that the plant hormone signal transduction pathway served key functions in the flower developmental stage under uniconazole treatment. Basing on the transcriptional analysis of genes involved in flower development, we hypothesized that uniconazole treatment increases the ratio of female flowers by activating the transcription of pistil-related genes. This phenomenon increases opportunities for pollination and fertilization, thereby enhancing the fruit-bearing rate. In addition, uniconazole treatment regulates the expression of unigenes involved in numerous transcription factor families, especially the bHLH and WRKY families. These findings suggest that the uniconazole-induced morphological changes in litchi inflorescences are related to the control of hormone signaling, the regulation of flowering genes, and the expression levels of various transcription factors. This study provides comprehensive inflorescence transcriptome data to elucidate the molecular mechanisms underlying the response of litchi flowers to uniconazole treatment and enumerates possible candidate genes that can be used to guide future research in controlling litchi flowering.

## Background

Litchi is an important tropical fruit widely cultivated in more than 20 countries in tropical and subtropical regions worldwide [1, 2]. In China, the planting area and total output of litchi are more than 0.59 million hectares and 1.91 million tons, respectively, according to the Agricultural Statistics Program of China [2]. However, the easily flowering and difficult fruit setting of litchi bring serious problems in its cultivation and production. Without treating or pruning, inflorescence development and flowering can consume excessive amounts of accumulated nutrients, thus leading to low fruit-setting percentage and even zero yield [3].

At present, mechanical and chemical methods of flower thinning have been employed in inflorescence control and management [3]. However, mechanical thinning is time consuming and labor intensive, and its effect is easily affected by temperature and weather [3]. Therefore, chemical flower thinning is preferred by litchi growers. Uniconazole (S-3307) is an important plant growth regulator widely used in inflorescence control because of its high efficiency, low toxicity, low residual, and low environmental pollution [4]. This chemical is a new plant growth retardant that can regulate numerous growth and development processes, such as flowering period [4, 5], controlcrop type [6, 7], enhance resistance [8, 9], and increase output and quality [10]. In *Arabidopsis*, treating shoots with uniconazole can result in enhanced primary root elongation and bolting delay [11], and it can also inhibit biosynthesis of gibberellins (GA) [11], trans-zeatin [12] and abscisic acid (ABA) [13]. Application of uniconazole can inhibit the flowering response induced by short-day treatment, and the inhibition by uniconazole is canceled by further application of GA1 in *Pharbitis nil* [14]. Moreover, uniconazole can effectively suppress excessive vegetative growth of soybean during the flowering stage, delay senescence of photosynthetically active leaves at pod-setting stage, and induce higher yield [15]. In litchi cultural areas, S-3307 spraying has become an important cultivation technique for flower control and fruit retention [4, 16]. S-3307 can significantly increase the fruit-setting rate to improve yield [16]. The present study compares and analyzes the transcriptional level differences between the treatment and control groups, and clarifies the metabolic pathways involved in litchi fruit setting.

## Materials and methods

### Plant materials and treatments

Six randomly selected 10-year-old litchi trees (*Litchi chinensis* Sonn. cv. Feizixiao) grown in an orchard in the South Subtropical Crops Research Institute (Zhanjiang, China) were selected, and 20 similar-sized inflorescence clusters from each tree were tagged. Three trees were treated with 50 ppm uniconazole when the length of inflorescence reached approximately 15 cm, and the remaining untreated trees were used as control. Each tree was treated as a biological replicate. Ten clusters in each tree were used to track the flowering and fruiting dynamics, and the others were used for sampling. The total flower number, female flower number, ratio of female to male flowers, fruit number, and fruiting rate were surveyed during the development period. The entire inflorescences of the uniconazole-treated and control trees were sampled at 28 days after treatment (DAT), immediately frozen in liquid nitrogen, and then stored at −80°C for future analysis. Three control spikes and three uniconazole-treated spikes at 28 DAT were respectively blended and pulverized to a mixed sample. These two mixed samples were used in transcriptome sequencing.

### RNA extraction and library construction

Total RNA was extracted from the inflorescence mixed sample in accordance with the method described by Zhang et al. [17]. The total RNA was purified with DNase I (Takara, Otsu, Japan) and RNase-free columns (Huayueyang, Beijing, China) [18]. RNA integrity and quality were assessed using agarose gel electrophoresis, NanoDrop ND-1000 (Thermo Scientific, Waltham, MA, USA), and Agilent 2100 Bioanalyzer (Agilent, Santa Clara, CA, USA). RNA (2 μg) was synthesized to cDNA using the RevertAid First Strand cDNA Synthesis Kit (Thermo Fisher, USA) through a one-step method. cDNA library construction and Illumina sequencing were performed using the Illumina HiSeq™ 2500 platform (San Diego, CA, USA) and then analyzed at the Beijing Genomics Institute (Shenzhen, China) as previously described [17, 19].

### *De novo* assembly and functional annotation

After raw reads were filtered to exclude low complexity reads, transcriptome *de novo* assembly was performed by using Trinity, a short-read assembling program [20]. For more details, sees S1 Fig. The resultant sequences obtained with Trinity are called unigenes. All assembled unigene sequences were aligned by BLASTX (*E*-value < 10^−5^) to public protein databases, the NCBI non-redundant protein (Nr) database, the Swiss-Prot protein database, the gene ontology (GO) database, the Kyoto Encyclopedia of Genes and Genomes (KEGG) database, and the Clusters of Orthologous Groups database [21]. To identify uniconazole-regulated genes, the threshold of differential unigene expression between the treated and control samples was set to FDR ≤ 0.05, |log_2_|≥ 1, and *P*-value < 0.01. The screened differentially expressed unigenes (DEGs) were further subjected to GO enrichment analysis and KEGG pathway enrichment analysis to verify biological significance [21, 22] (S1 File).

### Quantitative reverse transcription–polymerase chain reaction (qRT-PCR) analysis

qRT-PCR was performed on a LightCycler 480 II (Roche, Switzerland) using the SYBR green fluorescent label. cDNA was synthesized from total RNA using a PrimeScript RT Reagent Kit (Thermo Fisher, USA). The relative expression levels of genes were calculated using the 2^−ΔΔ*Ct*^ method. All quantitative PCRs were performed in three biological replications.

## Results and discussion

### Effects of uniconazole on flowering in litchi

**Fig 1:**
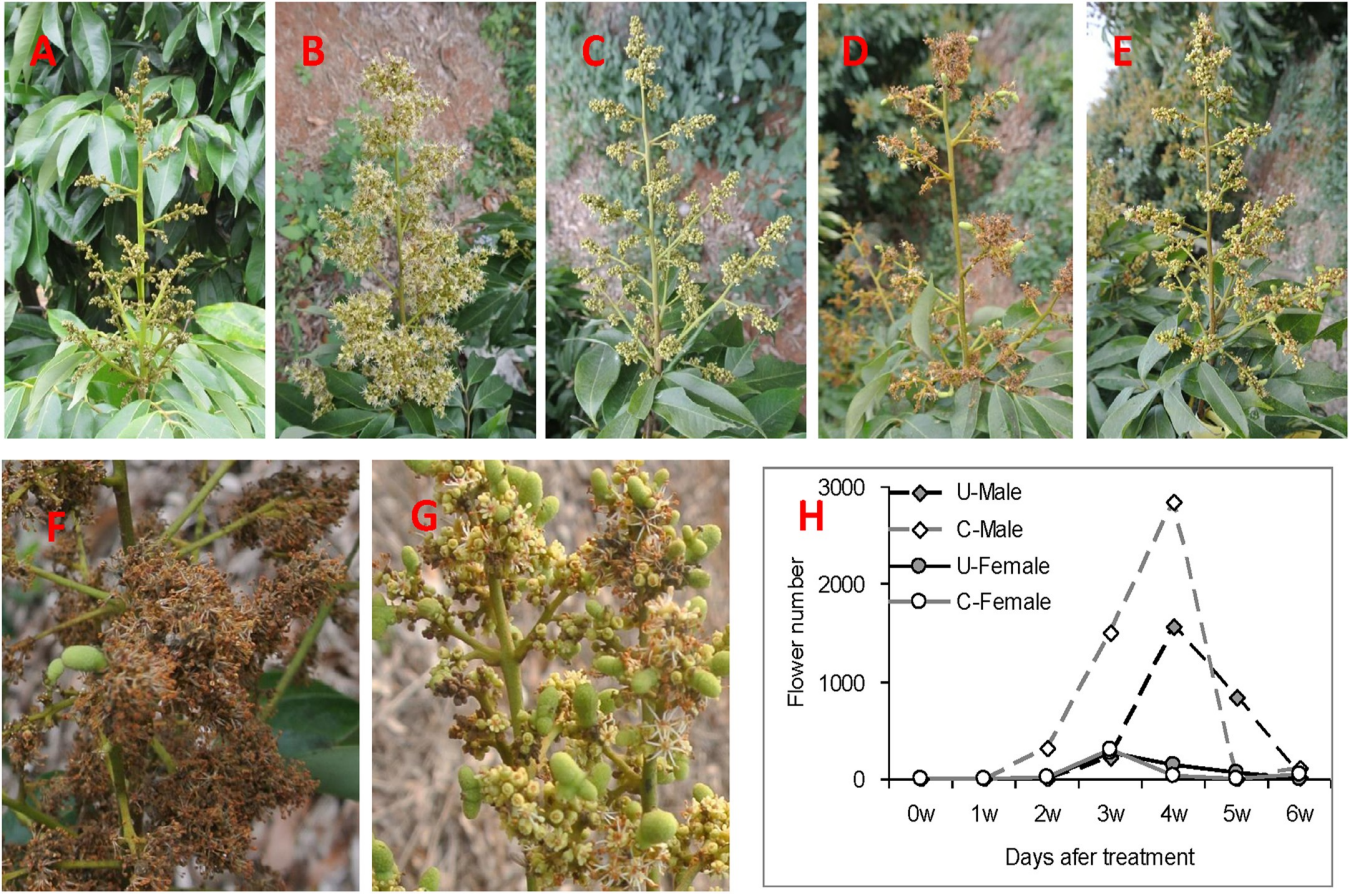
Effects of uniconazole on inflorescence development and flowering in litchi. (A) Inflorescence state before uniconazole application (30 days after floral evocation); (B) Inflorescence states from untreated control (B1) and uniconazole-treated (B2) plants four weeks after treatment (full-bloom stage of untreated control); (C) Clusters from untreated control (C1) and uniconazole-treated (C2) plants six weeks after treatment (fruitlet stage after abscission); (D) D1 and D2 represent the magnification of the portions of C1 and C2, respectively; (E) Effects of uniconazole on the blossoming process of male flower and female flower.

To ascertain the effects of uniconazole application on litchi blossoming and fruit bearing, we treated litchi inflorescence clusters with 50 ppm uniconazole and water (control) when the length of inflorescence reached approximately 15 cm (Fig 1A). As shown in Fig 1B and 1C, the flowers of water-treated inflorescences began to open at two weeks after treatment, whereas those of uniconazole-treated inflorescences remained closed, thus suggesting that uniconazole delayed flower development. Compared with the control, uniconazole treatment decreased the total flower number and male flower number and increased the number of females and ratio of female to male flowers (Fig 1H, Table 1). Subsequently, as expected, uniconazole application highly and significantly increased the number of fruits and fruiting rate per inflorescence (Fig 1D, 1E, 1F and 1G, Table 1).These results indicated that uniconazole treatment changed the flowering time of litchi and markedly increased the number of female flowers to improve fruit yield.

**Table 1:** Effects of uniconazole treatment on flowering and fruiting in litchi.

Treatment	Number of flowers/ Inflorescence	Number of females/Inflorescence	Ratio of female to male	Number of fruits/Cluster	Fruiting rate (%)
Uniconazole	3283.9±296.0*	534.1±48.3	19.40%**	5.7±0.7	1.1
Water	5016.0±445.6	349.9±64.8	7.50%	0.5±0.4	0.1

“*” and “**” indicate significant difference at P ≤0.05 and P ≤0.01, respectively.

### DEGs in response to uniconazole application on inflorescence

**Fig 2:**
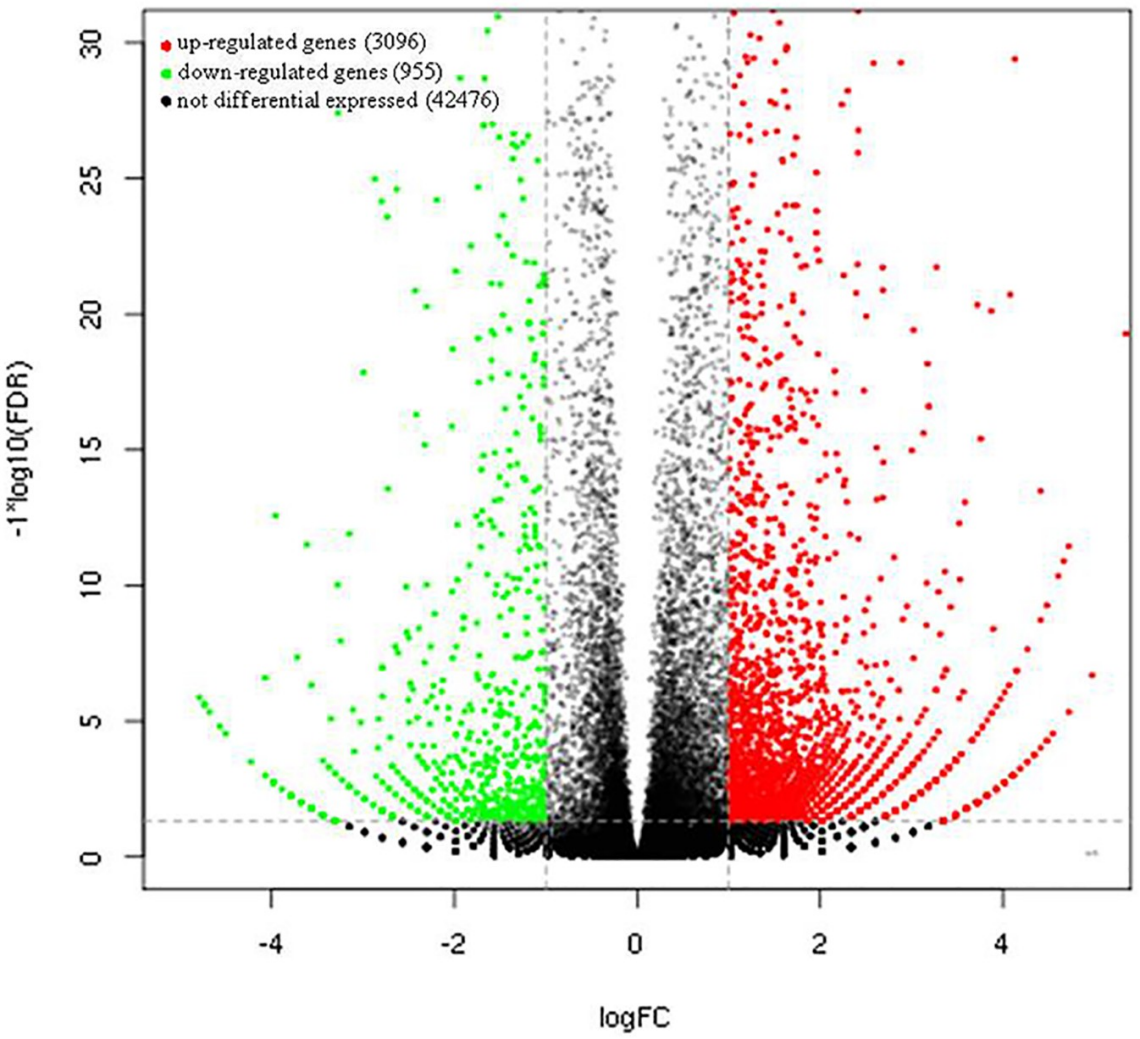
Volcano plots showing the comparison of DEGs between the treatment and control groups. The red scatters indicate upregulated DEGs, green scatters indicate downregulated DEGs, and black scatters indicate no DEGs between the uniconazole-treated and untreated samples. Datasets were filtered to remove genes with low expression levels (dotted line from −1 to 1 on the *x*-axis), and a significance cut off (*p*< 0.01) was applied (dotted line on the *y*-axis).

Differences in gene expression were assessed, and DEGs were identified by pairwise comparisons of the two libraries with the threshold of FDR ≤ 0.05 and |log_2_|≥ 1 (Fig 2). A total of 4051 DEGs (3096 upregulated and 955 downregulated) were identified in the pair-wise comparison between any two stages (S1 Table). The results showed that the number of upregulated DEGs was considerably greater than that of downregulated DEGs (Fig 2). The comparison of the expression levels for the uniconazole-treated and control groups is shown vividly in the volcano plots (Fig 2). These findings indicated significant differences in unigene expression at the flowering stages after uniconazole application.

### Functional analysis of DEGs in response to uniconazole application

**Fig 3:**
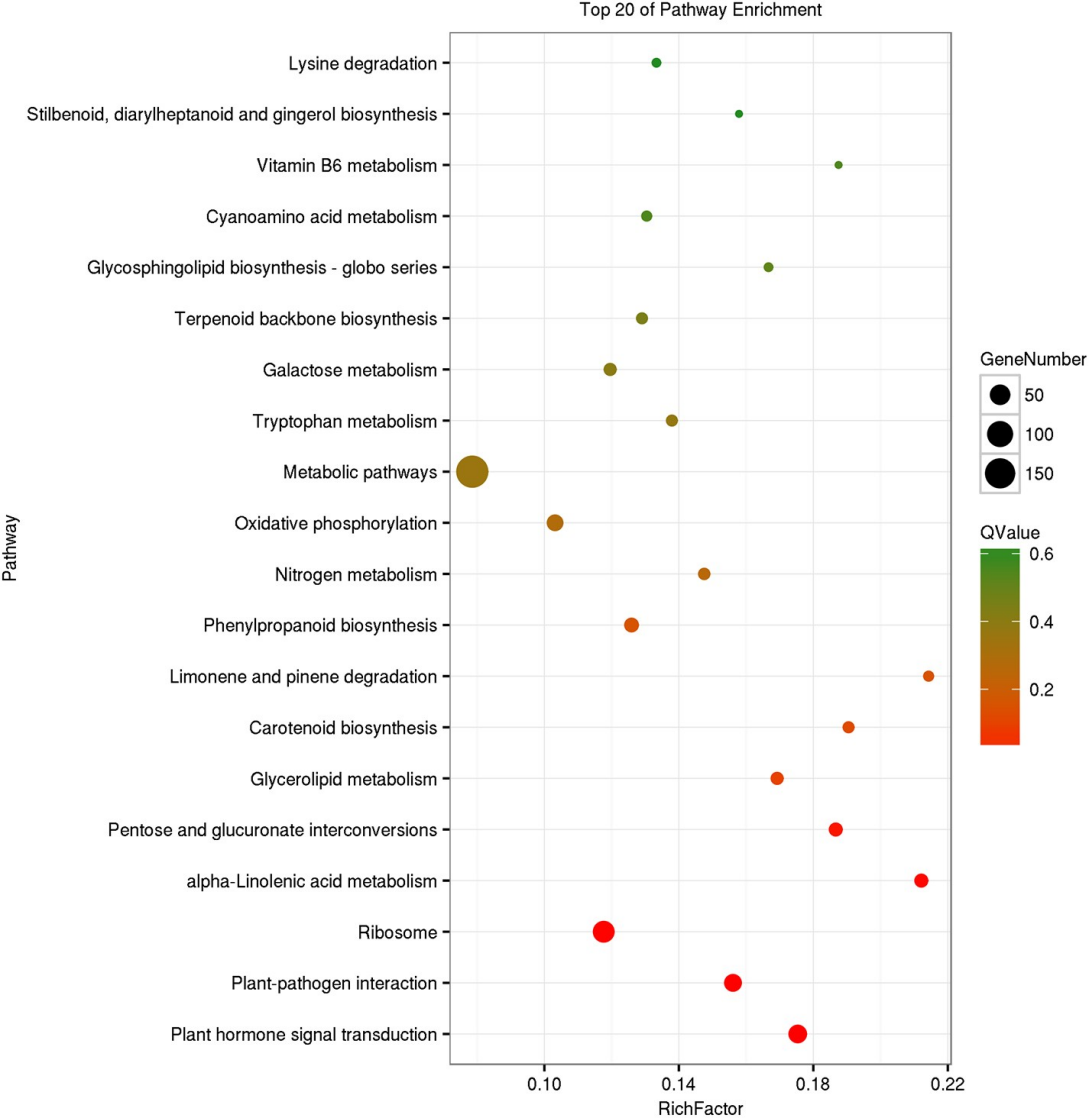
Functional analysis of DEGs based on the KEGG pathway. Pathways with a *Q*-value ≤ 0.05 significantly enriched in DEGs were analyzed in the comparison between treatment and control at the flowering stages. The degrees of KEGG enrichment can be measured by the richness factor, *Q*-value, and gene number enriched in this pathway. The right *y*-axis represents the KEGG pathway, and the *x*-axis shows the richness factor, which denotes the ratio of the number of DEGs to the number of annotated genes enriched in this pathway.

Enrichment studies of DEGs in GO and KEGG functional categories in pairwise comparisons were performed to evaluate the potential function of differentially expressed transcripts after the application of uniconazole. The detailed information of GO and KEGG is presented in S2 Table and Fig 3. GO functional enrichment indicated that 986, 525, and 1115 DEGs could be classified into three categories in GO assignments, namely, biological process, cellular component, and molecular function at the flowering stages, respectively (S2 Table). As shown in S2 Table, the top two categories were “calcium ion transport” and “pollen wall assembly” in the biological process category. With respect to the cellular component category, the majority of DEGs were involved in “intrinsic component of membrane” and “membrane part.” Under the molecular function category, “oxidoreductase activity” and “protein kinase activity” accounted for the major proportion.

The KEGG database was used to understand further the biological functions and pathways of DEGs. For the KEGG pathway enrichment analysis, pathways displaying significant changes (Q value ≤ 0.05) in response to uniconazole treatment were identified, with 591 DEGs categorized to 99 pathways at the flowering stages (S3 Table). The top 20 KEGG pathways compared are presented in Fig 3. The pathways involving the highest number of DEGs were “Metabolic pathways,” followed by “Biosynthesis of secondary metabolites” and “Ribosome,” indicating that these pathways are active at the flowering stage. The “plant hormone signal transduction” term was the most significantly enriched at the flowering stage (Fig 3). The high ratio of “plant hormone signal transduction” pathway implied that unigenes involved in the hormone signaling perform key functions at the flower developmental stage under uniconazole treatment. At the flowering stage, in addition to the “plant hormone signal transduction” pathway, the three hormone-related KEGG pathways “alpha-linolenic acid metabolism,” “carotenoid biosynthesis,” and “tryptophan metabolism” were also enriched. These results suggest that hormones serve crucial functions during this particular period (Fig 3).

### Transcriptional analysis of genes involved in the hormone signaling pathway

**Fig 4:**
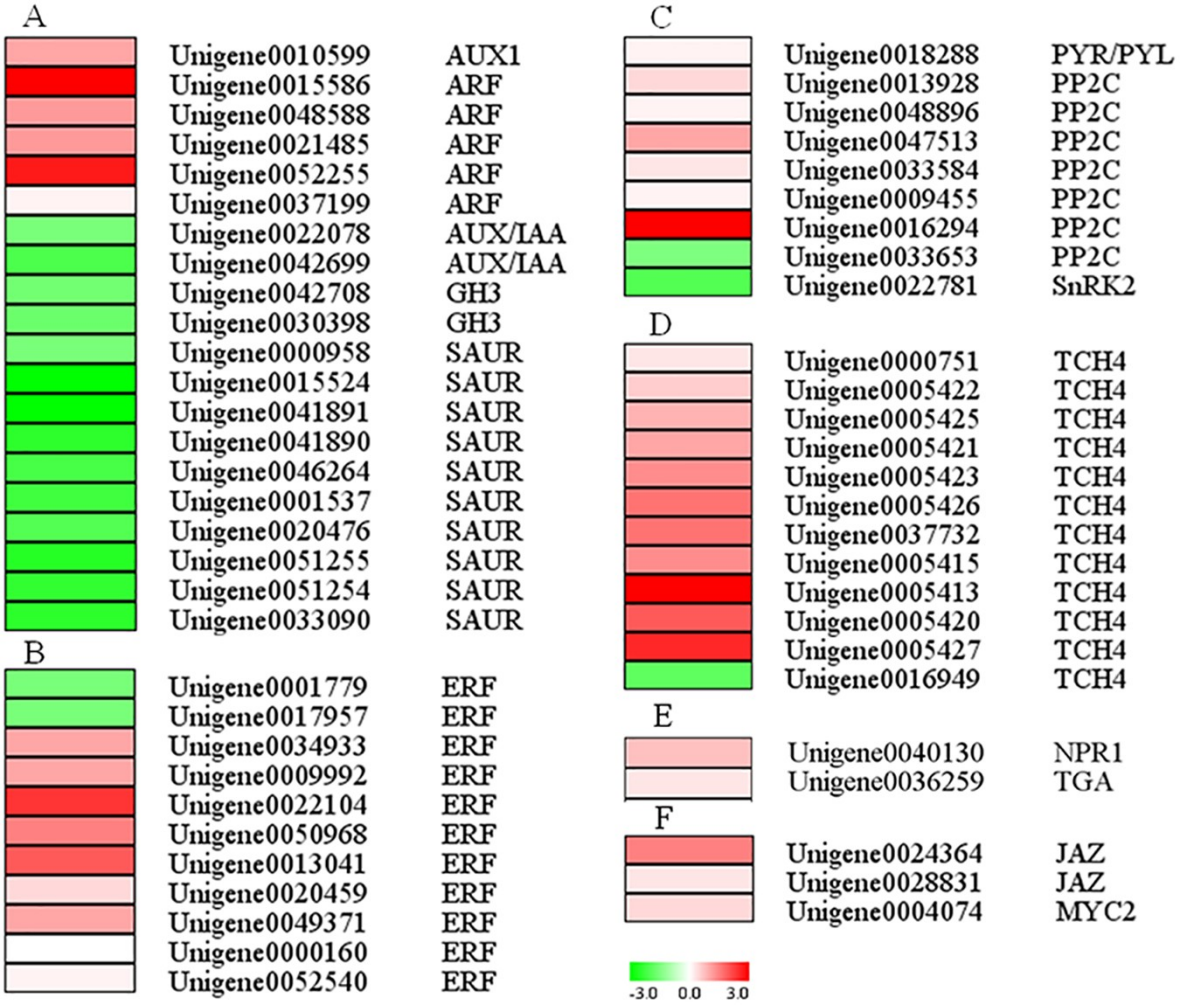
Heat map diagrams of relative expression levels of DEGs in the hormone signal transduction pathways. DEGs in the signal transduction pathways identified in KEGG pathway enrichment analysis are shown as auxin (A), ethylene (B), abscisic acid brassinosteroid (C), brassinosteroid (D), salicylic acid (E), and jasmonic acid (F). Ratios are expressed as log2 RPKM (treatment/control) values. Red and green colors indicate gene upregulation and downregulation, respectively.

For the KEGG enrichment analyses of the DEGs, the “plant hormone signal transduction” pathway was highlighted as being particularly affected at the flowering stage by uniconazole treatment (Fig 3). To understand the functions of plant hormones in inflorescence buds in response to uniconazole treatment, homologous genes involved in various hormonal regulation pathways in *Arabidopsis* were identified [23], and 57 unigenes predicted to be related to plant hormone signaling pathways were found to be differentially expressed at the flowering stage after uniconazole treatment (Fig 4, S4 Table), including those related to auxin (20 genes), ethylene (11 genes), abscisic acid (ABA, nine genes), brassinosteroid (BR, 12 genes), salicylic acid (SA, two genes), and jasmonic acid (JA, three genes). The result indicated that uniconazole treatment significantly affected the expression of genes involved in most plant hormones.

Among these genes, those encoding auxin comprised the largest group, and one unigene encoding auxin transporter protein (*AUX1*) and five unigenes encoding auxin response factor (*ARF*) were upregulated after uniconazole spraying. In comparison, the three families of early auxin responsive genes, auxin-induced proteins (*Aux/IAA*) (two genes), indole-3-aceticacid-amidosynthetase (*GH3*) (two genes), and SAUR family protein (*SAUR*) (10 genes) were significantly repressed at the flowering stage after uniconazole treatment (Fig 4A). In the ethylene-responsive pathway, seven out of the night genes encoding ethylene response factor (*ERF*) were upregulated after uniconazole treatment (Fig 4B). Seven unigenes annotated as protein phosphatase 2C (*PP2C*) involved in ABA signal transduction were differentially expressed, and six out of the seven genes were upregulated by uniconazole treatment (Fig 4C). As a plant growth retardant, uniconazole reduces the shoot growth of plants by inhibiting gibberellin biosynthesis. The increase in ethylene and active forms of cytokinins and the decrease in gibberellin in shoots may be the basis for the physiological phenomena caused by uniconazole [24]. Unexpectedly, gibberellin and cytokinin signaling pathways were not enriched, and related unigenes were barely expressed differently after uniconazole treatment. These findings suggest that the flowering of litchi affected by uniconazole may not occur through gibberellin and cytokinin. As a strong competitive inhibitor of ABA 8′-hydroxylase, uniconazole inhibits the ABA catabolism in *Arabidopsis* [13]. Hu [25] et al. found that uniconazole spraying could reduce the endogenous hormone GA, increase the hormone ABA, and change the flowering dynamics because of the change in endogenous hormones. Low GA concentration and high ABA promote female development, thereby significantly increasing the female rate [25]. Six *PP2Cs* involved in ABA signal transduction were markedly upregulated by uniconazole treatment (Fig 4C), thus suggesting that ABA could serve a positive function in affecting the female flower rate. Several studies showed that male flower development is closely associated with relatively high IAA contents and that low IAA concentrations induce female flower differentiation [26]. This study also found that the three families of early auxin responsive genes, *Aux/IAA*, *GH3*, and *SAUR*, were all downregulated after uniconazole treatment (Fig 4A). This result suggested that IAA exerted a negative effect on the female flower rate.

In addition to IAA, ABA, and ethylene, other hormones may also perform a function in uniconazole response. Among the unigenes related to BR signaling, the levels of 12 xyloglucan endotransglucosylase/hydrolase protein genes, annotated as *TCH4*, mostly increased after uniconazole treatment (Fig 4D). Genes related to SA and JA were also upregulated during the flowering process after uniconazole treatment. Moreover, the treatment upregulated one non-expressed or pathogenesis-related protein 1a gene and one BZIP transcription factor family protein gene (Fig 4E). Similarly, the treatment upregulated the jasmonate ZIM-domain protein gene and the*MYC2* gene (Fig 4F). The research outcomes indicated that uniconazole treatment significantly increased the expression levels of numerous key genes involved in the signal transduction of BR, SA, and JA. In particular, the expression levels of most TCH4s markedly increased, thus implying that hormones BR, SA, and JA could be involved in the response to uniconazole treatment. At present, however, related studies on the response of hormones BR, SA, and JA to uniconazole are lacking. Combined with those of previous studies, our results suggest that diverse hormonal signals are involved in uniconazole responses and that these hormones may jointly regulate the development of litchi flowers after uniconazole treatment. However, the exact functions of multiple hormones in this process still require further investigation.

### Transcriptional analysis of genes related to flower development and sex determination in litchi

Uniconazole treatment significantly affected the flowering characters of litchi, which consequently increased fruit yield (Fig 1, Table 1). As one of our aims was to identify the genes responsible for flower development and sex determination, 18 homologous DEGs involved in flower development in *Arabidopsis* were specifically searched from the two libraries (Table 2). Among these genes are those that control floral organ identity [ABC model genes (*AP2*), stem cell maintenance (*CLAVATA1*,*CLV1*), pistil development [(*CLAVATA3*, (*CLV3*), *LEAFY*, (*LFY*), *LEUNIG*, (*LUG*)], and flowering time [*TERMINAL FLOWER 1*, (*TFL1*), *FLOWERING LOCUS T* (*FT*), *FLOWERING-PROMOTING FACTOR 1* (*FPF1*), and other MADS-box genes (*SQUAMOSA PROMOTER BINDING-LIKE 8*, *SPL8*)] (Table 2).

**Table 2:** Expression analysis of genes related to flowering in inflorescence buds of litchi after uniconazole treatment.

Gene ID	CK2_rpkm	U2_rpkm	log2 Ratio(U2/CK2)	Abbreviation	Annotation
Unigene0002425	1.66	5.05	1.60	AP2	AP2/ERF domain transcription factor [*Medicagotruncatula*]
Unigene0009862	2.57	0.79	−1.70	AP2	AP2 domain-containing transcription factor [*Theobroma cacao*]
Unigene0032409	36.04	16.81	−1.10	AP2	AP2 domain-containing transcription factor [*Populustrichocarpa*]
Unigene0007683	0.88	1.97	1.16	AP2	AP2 domain-containing transcription factor [*Theobroma cacao*]
Unigene0007332	0.30	2.27	2.93	AP2	AP2/ERF and B3 domain-containing transcription repressor [*Populuseuphratica*]
Unigene0013522	1.34	3.93	1.56	AP2	AP2/ERF and B3 domain-containing transcription factor [*Citrus sinensis*]
Unigene0046187	2.73	1.16	−1.24	CLV1	CLAVATA 1-like [*Citrus sinensis*]
Unigene0017753	8.08	3.79	−1.09	CLV3	CLAVATA 3 [*Pyrus x bretschneideri*]
Unigene0020360	2.11	4.55	1.11	CLV3	CLAVATA 3 [*Nelumbonucifera*]
Unigene0039839	1.32	3.33	1.34	LFY	LEAFY [*Litchi chinensis*]
Unigene0025554	0.50	1.92	1.95	LUG	LEUNIG [*Citrus sinensis*]
Unigene0015457	0.00	1.34	10.39	LUG	LEUNIG [*Pyrus x bretschneideri*]
Unigene0040635	13.62	28.18	1.05	SPL	SQUAMOSA PROMOTER BINDING-LIKE 8 [*Theobroma cacao*]
Unigene0028669	3.29	6.67	1.02	TFL1	TERMINAL FLOWER 1 [*Dimocarpuslongan*]
Unigene0032646	0.92	5.16	2.49	TFL1	TERMINAL FLOWER 1 [*Dimocarpuslongan*]
Unigene0032778	3.33	1.14	−1.54	FT	FLOWERING LOCUS T [*Litchi chinensis*]
Unigene0032779	0.12	1.57	3.72	FT	FLOWERING LOCUS T [*Litchi chinensis*]
Unigene0038863	5.66	1.00	−2.50	FPF1	FLOWERING-PROMOTING FACTOR 1-like [*Citrus sinensis*]

As shown in Fig 1 and Table 1, uniconazole treatment significantly increased the ratio of female to male flowers, thus indicating that uniconazole could affect the differentiation of male and female flowers in litchi. Interestingly, several pistil-related genes were differently expressed after uniconazole treatment. *LUG*, which is a critical regulator of gynoecium marginal tissue development [27], negatively regulates *AGAMOUS* expression in the first two whorls of the *Arabidopsis* flower [28]. Two *LUG* genes were highly induced by uniconazole treatment (Table 2), which increased the ratio of female to male flowers. This result suggests that LUG genes play similar roles during litchi flower development. As shown in Table 2, one *CLV1* gene and two *CLV3* genes were differently expressed. Genetic analysis indicates that *CLV1* acts with *CLV3* to control the balance between meristem cell proliferation and differentiation. The *CLV3* gene is expressed in the putative stem cells at the apex of shoot, floral, and axillary meristems [29]. These results suggest that uniconazole treatment activates pistil development by affecting the expression of pistil-related genes in litchi.

Among the floral organ identity genes, six orthologs of *AP2*, an A-class gene in *Arabidopsis* [30], were identified, and four orthologs were upregulated after uniconazole treatment. *AP2* belongs to the *AP2/ERF* family, which contributes to the formation of the floral meristem and floral organ, and interacts with floral key genes *AP1* and *LFY* [31–33].

Aside from significantly improving the ratio of female to male flowers, uniconazole spraying could delay floral organogenesis and flowering time (Fig 1). Uniconazole treatment of inflorescence buds upregulated two orthologous genes of *TFL1* (Table 2). *TFL1*, a floral repressor in *Arabidopsis*, regulates flowering time and maintains the fate of inflorescence meristem. The *tfl1-1* mutation causes early flowering and limits the development of the normally indeterminate inflorescence by promoting the formation of a terminal floral meristem [34]. The result implies that uniconazole treatment could activate the expression of *TFL1* genes, which agrees with our observation that the flowering time of the uniconazole-treated inflorescences was later than that of the control inflorescences (Fig 1). One putative homolog of *SPL8* was differently expressed at the flowering stage. The SBP-box gene family regulates diverse aspects of plant development, especially flower development. In *Arabidopsis*, *SBP1* and *SBP2* participate in flower development by interacting with the floral meristem identity gene *SQUAMOSA* [35]. The *AtSPL3* gene has shown activity primarily in the inflorescence apical meristems, floral meristems, and floral organ primordial, and promotes floral transition [36, 37]. *AtSPL8* contributes to the formation of the sporangium and plant reproduction [38] and may also be involved in the GA signal transduction pathway [38]. *AtSPL9* can promote the transcription of floral genes *FUL*, *SOC1*, and *AGL42* [39].

### Uniconazole-responsive transcription factors

**Fig 5:**
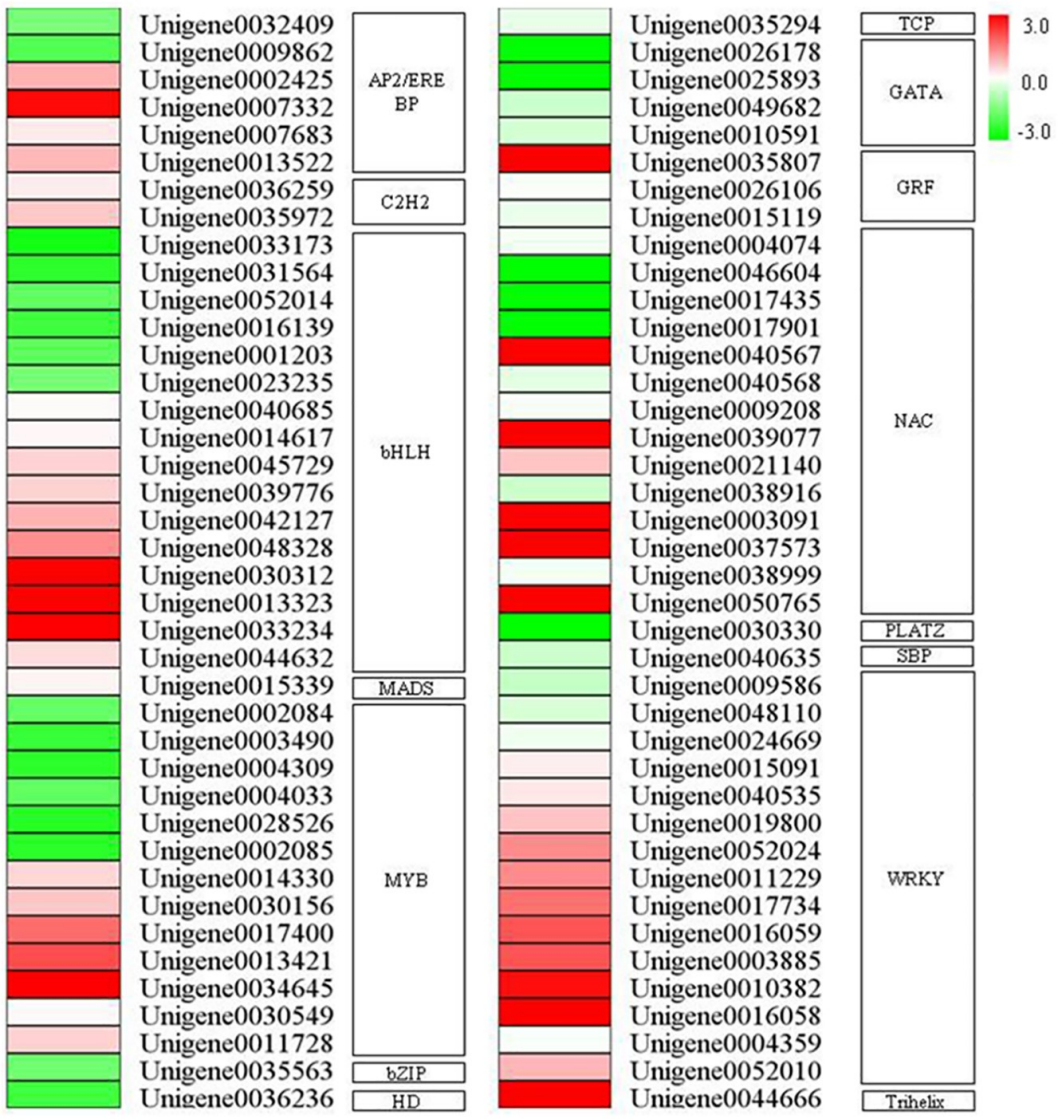
Differentially expressed genes encoding transcription factors following uniconazole treatment. Different shades of red and green express the extent of change according to the color bar provided in Fig 4. Red and green indicate upregulation and downregulation of genes, respectively, whereas white indicates that no change was detected after uniconazole treatment.

Previous studies demonstrated that several transcription factors may be master regulators of downstream effects and perform crucial functions in plant growth and development [40]. Sixteen putative transcription factor families (80 members) were identified in the flowers of litchi in response to uniconazole treatment, with 58 upregulated and 22 downregulated differentially expressed uniconazole-responsive transcription factors at the flowering phase (Fig 5, S5 Table). Among the transcription factor families, the largest ones were the bHLH family (16, 20.0%), followed by the WRKY (15, 118.8%), NAC (13, 16.3%), and MYB families (13, 16.3%) (Fig 5).

The bHLH family includes genes regulating diverse processes of flower development. Four bHLH transcription factors (*ALC*, *AMS*, *DYT1*, and *SPT*) controlling the development of flower have been cloned in *Arabidopsis thaliana*. *SPT* and *ALC* may be relevant to pistil development [41,42], but *AMS* and *DYT1* are closely related to the morphogenesis of anthers [43,44]. In the present study, as the largest transcription factor family, 16 unigenes with bHLH-like sequences were differentially expressed (10 upregulated and six downregulated) after uniconazole treatment. Such prevalence of bHLH transcripts in floral development implies that this family is as involved in flower development in litchi as it is in other species [45].

Putative homologs of WRKY transcription factors were significantly represented after uniconazole treatment, with 15 unigenes showing upregulated expression. WRKY transcription factors are mainly involved in biotic and abiotic stress responses [46] and senescence [47, 48]. In recent years, however, several members of this family have also been associated with floral development and flowering time in plants [46, 49–51]. *AtWRKY2* and *AtWRKY34* regulate pollen and pollen tube development [39]. *AtWRKY71* accelerates flowering via the direct activation of *FT* and *LFY* in *A*.*thaliana* [50]. *AtWRKY53* overexpression exerts the early flower phenotype [46]. *GsWRKY20* promotes the flowering of *Glycine soja* [52]. Two WRKY transcription factors, *OsWRKY11*and *OsWRKY72*, are involved in the determination of flowering time of rice [51, 53]. Significantly increased expression levels of all WRKY transcription factors after uniconazole treatment indicate that these transcription factors serve important functions in uniconazole-induced flower formation. However, the mechanisms by which WRKY transcription factors are involved in this process need further investigation.

The NAC transcription factor family was significantly expressed after uniconazole treatment, with 10 unigenes highly upregulated and 3unigenes downregulated. Several NAC members are involved in flower-boundary morphogenesis [54–56]. NST1 and NST2 participate in the formation of secondary anther walls [57]. *NTL8* and *LOV1* are implicated in regulating the flowering time of *Arabidopsis* [58, 59]. The MYB transcription factors have been identified as floral developmental regulators. *MYB21*, *MYB24*, and *MYB57* reportedly mediate the stamen filament growth of *Arabidopsis* [60, 61]. *AtMYB33* may mediate flowering by binding to the LEAFY promoter [62] and redundantly control anther development with *AtMYB65* [63]. In the present study, 13 putative MYB transcription factors were differently expressed after uniconazole spraying. These results indicate that the NAC and MYB transcription factors serve similar functions in controlling flower bud development in litchi and other species.

Several other transcription factor families were also found. Interestingly, DEGs belonging to the C2H2, MADS, TCP, GRF, MYC, SBP, and Trihelix families were upregulated after uniconazole treatment. By comparison, DEGs of the bZIP, HD, and PLATZ families were downregulated after uniconazole treatment. These results suggest that these transcription factors perform specific functions in uniconazole-induced flower formation. In addition, uniconazole-induced morphological changes in litchi inflorescences may be related to the expression levels of various transcription factors.

### RT-qPCR validation of DEGs from RNA-Seq

**Fig 6:**
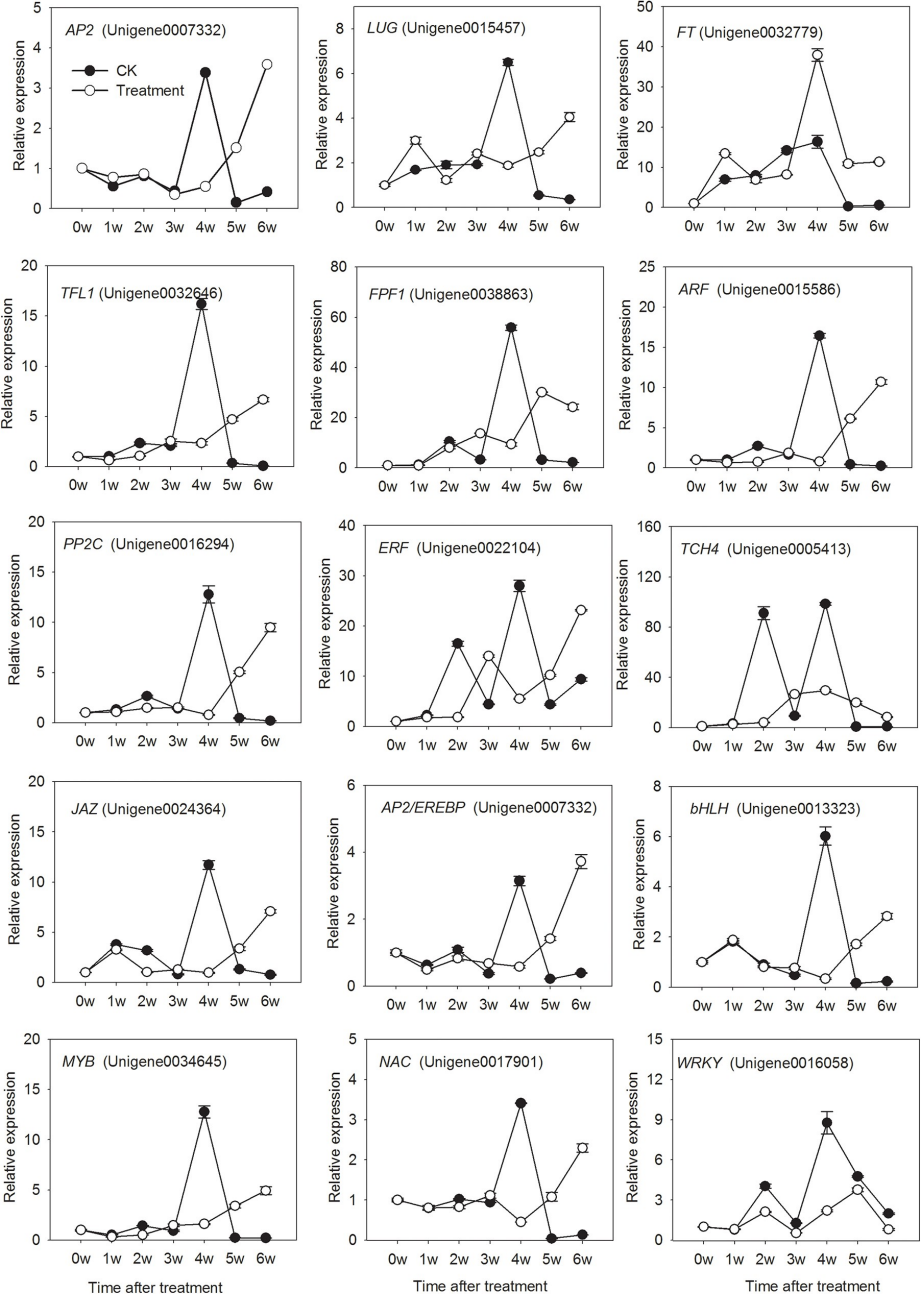
Verification of RNA-seq results by RT-qPCR. Transcript levels of 15 genes, including 5 probable transcription factors, 5 flowering-related genes, and 5 genes involved in hormone signaling, as measured by RT-qPCR analyses. The relative RT-qPCR expression is shown on the *y*-axis to the left, with error bars representing the standard error (*n* = 3).

To validate further our RNA-seq expression profile data, we performed RT-qPCR assays on 15 unigenes involved in hormone signaling and flowering, as well as on transcription factors related to uniconazole responses (Fig 6, S2 File). Fig 6 shows that the expression trends of these unigenes are in accordance with the prediction by RPKM value. The results validate the fact that the predicted unigenes related to hormone signaling, flowering genes, and transcription factors influence flowering characteristics under uniconazole induction.

## Conclusion

The pre-bloom application of uniconazole to “Feizixiao” litchi inflorescences delayed the flowering time and markedly increased the number of female flowers to improve fruit yield. This study provided a global expression profile of uniconazole-treated and untreated litchi inflorescences through the *de novo* assembly of next-generation sequencing. A total of 4051 DEGs were identified in response to uniconazole treatment, and further analysis indicated that the uniconazole response was complex. Uniconazole-induced changes in the morphology of litchi inflorescences could be related to the regulation of hormone signaling, flowering-related genes, and various transcription factors. These findings provide a platform for understanding the DEGs and pathways induced by uniconazole. This study will be useful for further studies on the response of flowering to uniconazole in litchi.

## Supporting information

S1 Fig Schematics of the transcriptome sequencing analysis in litchi. (DOCX)

S1 Table DEGs between any two stages. (XLSX)

S2 Table GO functional categories in pairwise comparisons. (XLSX)

S3 Table KEGG functional categories in pairwise comparisons. (XLSX)

S4 Table Differentially expressed genes at the flowering stage after uniconazole treatment. (XLSX)

S5 Table Differentially expressed uniconazole-responsive transcription factors at the flowering phase. (XLSX)

S1 File Gene ontology functional enrichment analysis for DEGs and KEGG pathway analysis. (DOC)

S2 File Fifteen unigenes related to uniconazole responses used to RT-qPCR assays. (DOCX)
