# Supplementary material for: Transcriptional changes in litchi (Litchi chinensis Sonn.) inflorescences treated with uniconazole
Source: PLoS One. 2017 Apr 18;12(4):e0176053. doi: 10.1371/journal.pone.0176053 (PMC5395186; doi:10.1371/journal.pone.0176053)
Supplement: S1 File — (DOC) [file pone.0176053.s007.doc]

**1. Gene Ontology Functional Enrichment Analysis for DEGs**

Gene Ontology (GO) is an international standardized gene functional classification system which offers a dynamic-updated controlled vocabulary and a strictly defined concept to comprehensively describe properties of genes and their products in any organism. GO has three ontologies: molecular function, cellular component and biological process. The basic unit of GO is GO-term. Every GO-term belongs to a type of ontology.

GO functional analysis provides GO functional classification annotation for DEGs as well as GO functional enrichment analysis for DEGs.

First, mapping all differentially expression genes to each term of Gene Ontology database (http://www.geneontology.org/) and calculating the gene numbers each GO term has. We get a gene list and gene numbers for every certain GO term, then using hypergeometric test to find significantly enriched GO terms in DEGs comparing to the genome background. The p-value calculating formula in this hypothesis test is:
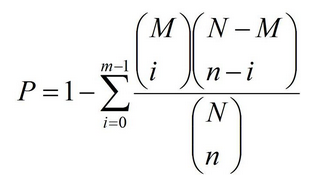


Where N is the number of all genes with GO annotation; n is the number of DEGs in N; M is the number of all genes that are annotated to the certain GO terms; m is the number of DEGs in M. The calculated p-value goes through Bonferroni Correction, taking corrected-p-value ≤ 0.05 as a threshold. GO terms fulfilling this condition are defined as significantly enriched GO terms in DEGs. This analysis is able to recognize the main biological functions that DEGs exercise.

Our GO functional enrichment analysis also integrates the clustering analysis of expression patterns. Thus, researchers can easily get the expression patterns of DEGs annotated to the given GO-term. For example, Immune system process is the most significantly enriched GO term in DEGs. Figure 4 shows the expression patterns of the involved DEGs (where Ratio represents the ratio of gene RPKM between samples).

**2. KEGG Pathway Analysis**

Different genes usually cooperate with each other to exercise their biological functions. Pathway-based analysis helps to further understand genes biological functions. KEGG is the major public pathway-related database. Pathway enrichment analysis identifies significantly enriched metabolic pathways or signal transduction pathways in DEGs comparing with the whole genome background. The calculating formula of p-value is similar with that in GO analysis. Here N is the number of all genes that with KEGG annotation, n is the number of DEGs in N, M is the number of all genes annotated to specific pathways, and m is number of DEGs in M.
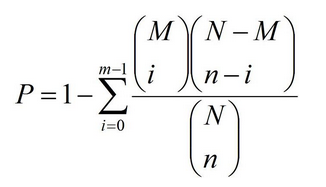


After multiple testing correction，we choose pathways with Q-value≤0.05 are significantly enriched in DEGs. With Pathway significantly enrichment, we can the main biochemical pathways and signal transduction pathways which DEGs take part in.
