# Supplementary material for: Transcriptional changes in litchi (Litchi chinensis Sonn.) inflorescences treated with uniconazole
Source: PLoS One. 2017 Apr 18;12(4):e0176053. doi: 10.1371/journal.pone.0176053 (PMC5395186; doi:10.1371/journal.pone.0176053)
Supplement: S2 File — (DOCX) [file pone.0176053.s008.docx]

**>Unigene0007332** 348 PREDICTED: AP2/ERF and B3 domain-containing transcription repressor RAV2-like [Populus euphratica]

TTCTCAAAACAACTGTCTAATACAGACGTCGAAAATAGACTAGCCATCCCCACGGTGAACTTGTGTGACTTTCCAGCAATCCCTGAAGGAGAGAATCACGTATTTGTTCCAGTCATGGATGAAGCTGGGCGGGAATGGAATTTTCGTCTTTCAAAAAGACGAGAAAGTAACTTCCTTAAACCTATTATTAGTTCTACAGATTGGAACCGTTATGTCAGAGAGAACCAGCTTCGTCAAGGTGATACGATCACTTTTTATAGGGAGAACGGTGAAGCTAATGGGGAGATTATCAGAATCAAAGTC

**>Unigene0015457** 270 PREDICTED: transcriptional corepressor LEUNIG-like [Pyrus x bretschneideri]

CTATGCCGTTGCATGGTTAACTCACAAAGTTTATTGTGGCAACCAAACTTTTCTTTGCATGCTGCACTCAAATGTCCCATAATGTCCCTCGACTTCCACCCTAAGAAGAACAATCTTTTTTGTTTTTGTGATAATGACCGTGAAATTCAGTATTGGAATATCAATCCATTATCATGCAGTCGTATCTCCAAGGTTGGC

**>Unigene0032646** 548 terminal flower 1 protein [Dimocarpus longan]

ATGGAAGCAAAATTGTCAGAGCCTCTTGTGGTTGGAAAAGTAGTTGGAGATGTTGTTGATTATTTCAATCCAACTGTGAAAATGACAATCATCTATAACTCCATGAAGCAAGTATATAATGGCCATGAGCTTTTTCCCTCAGCTGTGACTCAGAAACCCAAGGTTGAGGTTCATGGAGGTGATTTGAGATCTTTTTTCACTCTGGTCATGACTGACCCAGATGTTCCTGGTCCAAGTGACCCATACTTGAGAGAGCATTTACACTGGATAGTGACAGACATCCCAGGCACAACAGATGTCACGTTTGGAAAGGAGGCTGTGAGCTATGAAATTCCAAGGCCAAACATAGGGATTCACAGGTTTGTGTTTCTTCTCTTCAAGCAGAAACGAAGGCAGACAGTTACCAACTTACCATCTTCAAGAGATTGCTTTAACACTAGAAACTTTGCTGAAGAAAATGAGCTTGGTCTTCCTGTGGCTGCTGTTTTCTTCAATGCACAACGGGAAACAGCCGCAAGAAGACGC

**>Unigene0032779** 450 flowering locus T [Litchi chinensis]

ACAAAGTCAATTTCTCTTAGTGTTTCCTATAACAACAGGGAAATCAACAATGGTTGTGAGCTCAAACCCTCTCAGATTGTCAACCAACCTAGGGTTGATGTTGGTGGTGATGATCTGAGGACCTGCTACACTTTGGTTATGGTGGATCCTGATGCACCTAGCCCAAGTGAACCAAGCCTGAGGGAGTATTTGCATTGGTTGGTGACTGATATTCCAGCAACCACTGGGGCAACCTTTGGGCAAGAGATCGTGAGTTACGAGAGCCCCAGACCAACTGTGGGGATTCACAGGTTCGTATTCGTGTTGTTCCAGCAACCGAGCAGGCAGACTATGTATGCACCGGGGTGGCGCCAGAATTTCAACACCAAAGACTTTGCTGAACTTTACAACCTTGGATCACCGGTGGCCGCCGTCTACTTCAACAGCCAGAGGGAGAGTGGCTCCGGTGGA

**>Unigene0038863** 366 PREDICTED: flowering-promoting factor 1-like protein 3-like [Citrus sinensis]

ATGGCTGGTGTTTGGGTATTCAGGACCAACGGTGTGGTTCAACTGGAGAACCCATCTAGGCATAAGGTGCTGGTGCATGTTCCAACGAACGAGGTGATAAATTCTTACTCTGATCTTGAACGGATGCTGTCGTCTCTCGGATGGGAGAGGTACTACGAGGACCCGGATCTTCTTCAATTCCATAAACGATCCACTGTCCATCTAATCTCTCTCCCAAAAGACTTCAACAAGTTCAAGTCCGTGCACATGTACGACATCGTTGTCAAGAATCGTAATGTCTTCGAAGTTAGAGAC

**>Unigene0015586** 250 PREDICTED: auxin response factor 18 [Fragaria vesca subsp. vesca]

GACGATGATATGTTCTCTAAGGGTGAGAATGGTGAATTAGGAGTTGGCACTCAGCGAATTGCTCGTCAGCAGAGCCCAATACCTTCATCTGTGATATCCAGCCATAGCATGCATTTAGGAGTCCTTGTGACTGCTTCCCATGTTGTTATGACTGTCTCAGTGGGTGTT

**>Unigene0016294** 237 PREDICTED: probable protein phosphatase 2C 14 [Vitis vinifera]

GATGTCCATGACTGGCTCTTTAATCCTGGTTTAAGACATGCTTTGTTTATATTCAAACCAATTATGTCATCATCATCGTCTCTTTCCTCACTATCAACATCTTCATCATCATCACCACCATTCTCATCATTGACATCAACACCACTATCTTCTCAGACAGGAAGTTGTGGTTCAATTGAAGGGCTAAATGCAGGTGGGTCATCTGAGTTTTGTTTGTTTCTCTATGCCTGGAAGGTA

**>Unigene0022104** 565 PREDICTED: ethylene-responsive transcription factor ERF023-like [Citrus sinensis]

CAAGAATCAGCTCCACTCCAGTCCGACCCATCAACTCCAAAACCCGGCTTTAAGAGGAAGAGGCCCCGCCAGACCACCCAGTTGACGTTTCACGGCGTCCGCCAACGGAGATGGGGTAGTTATGTCTCCGAGATACGGTTACCGGGTCAAAAGACCAGGATATGGCTCGGTTCATTCGGCTCACCTGAGATGGCTGCCCGGGCATATGACTCCGCTGCCTTCTTCTTGAAAGGCAACTCAGCCGCGCTCAACTTCCCCGACTCGGCCGGATCGCTTCCCCGGCCAGAGTCATCTTCCCGGAGAGACATTCAGCTGGCGGCGGCCAAAGCGGCGGCGGTGGTTCAGAATCAAAAGACGGCTAGCAACAGGGTCGAGTCTGGTAGTGAACCGGAGAGTGAAAGTTGCCAGGAAGCGATCACGTTTGAGCAAATGAAGAGGAACCCCCTGATGAGCCCACTTAGGTTTGACTCAGATGTTGGAGAGTTGTATTGTTTTATGGATGATGATGAGTTCCTAGTTGGGTCTTGTTTTGAATTT

**>Unigene0005413** 116 PREDICTED: probable xyloglucan endotransglucosylase/hydrolase protein 23-like [Glycine max]

AACTATATGATCTACAATTACTGCACTGACTCAAAGAGATTTCCACAGGGAATCCCCCCAGAATGCAAGGCCTCCTAAACTGAGTTAAAGTTATTACATGTACAATTCATT

**>Unigene0024364** 736 PREDICTED: protein TIFY 9-like isoform X1 [Citrus sinensis]

ATGTCGAGAGCCACCATGGAGCTCGATTTCTTTGAAATGGAGAAGAAGGAGAACTCCTCCTCCTCCAACCGGTCTCAGTTCAAGAAATTCCTCCGTCATCAGAGAAGCTTTCGAGATATTCAAGGCGCCATTTCGAAGATAAATCCGGAGATACTCAAATCTGTGATCGCGTCCGGTTCTGTGAATACGGACAACGGTAACACGTTCTCTTTGCCGTCTACTCCTAAGGAAGATCCGGTGCCTCTCCCGGCGCTGCCTCTTTATAGACACATTCCAAGGCCTGCTCCAGAAAACCTCCCCGAAACGGCTCCTCTGACCATTTTCTACAACGGAACCGTCACCGTTTTCGACGTTCCTCGAGACAAGGCTGAGACGATACTGAAACTTGCTCTGGAAGGAAACTCCAACATTGCGGAGTCAAATCAGCAACAGCTGCTGGAGACACTCAACAATGGAGGTCAGTCAATCAGTCAGTCGGTCAATCTTTTGTGTTTTAACATAAAAGTCGTTTGATTGTTGACTGGTTTTGAAAATGTTACTGGAATTTGAATTTTGCGGTGGTTTGCAGATCTGCCCCTTGCTCGTAGAAAGTCGTTGCAGAGATTCCTCGAGAAGCGCAAGGAGAGG

**>Unigene0007332** 348 PREDICTED: AP2/ERF and B3 domain-containing transcription repressor RAV2-like [Populus euphratica]

TTCTCAAAACAACTGTCTAATACAGACGTCGAAAATAGACTAGCCATCCCCACGGTGAACTTGTGTGACTTTCCAGCAATCCCTGAAGGAGAGAATCACGTATTTGTTCCAGTCATGGATGAAGCTGGGCGGGAATGGAATTTTCGTCTTTCAAAAAGACGAGAAAGTAACTTCCTTAAACCTATTATTAGTTCTACAGATTGGAACCGTTATGTCAGAGAGAACCAGCTTCGTCAAGGTGATACGATCACTTTTTATAGGGAGAACGGTGAAGCTAATGGGGAGATTATCAGAATCAAAGTC

**>Unigene0013323** 410 PREDICTED: transcription factor bHLH66-like isoform X4 [Vitis vinifera]

CGCTTCATTTTCTCTACCATTTTTTCCTTTCTTTCAACCCAACGAACCCTAAATCCCTTCCTAACTTGACTTCTTTGCTTTGCTTGTTTAGGTATGGAAGGACAGCGGAACCTGGAGATGTCTTTGAACATTGGTGGGAATGCCAATTATGTCTCTTGTCGACCTGAAGATGATCCAAATTGGCACTTTGTCATGGGAACTGGAAGGAGTGGGGATCAACCTAACGCTCTGTCGTCTTCACCTCAATGGACCACCCACTTACAGGAAGCACCTCTCAATTATGTTGAATTTCTTGCTGAAAATTCCACGTTTCTTGAGAACGCTTCTGGTGCAGACATGTTAGAGACCATTACTTCAACTGTAGGTGGCATGCAATCTGTTGAGGGACTCTCTGAAATCCAAAAAGAA

**>Unigene0034645** 225 PREDICTED: transcription factor MYB48 [Vitis vinifera]

TGCATTGGTGAGGCTCAGTTACAGCTTTCATTCCCACAAGTCCTCCCGCAACCCCAACAAGACATGACATTGTCACCAGGAAGCCAAGAACTCTTGTCTAGGTTCGGGGATCCTTATTTTTTCGACGTGTTCGGGCCAGTGGATGATGCTATCGAGCTTGCT

**>Unigene0017901** 1289 NAC domain protein, IPR003441 isoform 1 [Theobroma cacao]

ATGAATTCATTTTCACACGTTCCACCGGGCTTCAGATTCCATCCCACTGATGAAGAACTAGTTGATTACTACCTGAGAAAAAAAGTTGCTTCGAAAAGGATTGATCTAGATGTCATCAAAGATGTTGACCTTTACAAAATTGAGCCATGGGATCTTCAAGAGTTGTGCAAACTAGAAACTGAAGAGCAGAATGAATGGTATTTCTTTAGCCACAAAGATAAGAAATACCCAACAGGAACTCGCACAAATAGAGCAACCAAAGCTGGGTTTTGGAAAGCCACAGGCAGGGATAAAGCTATCTACTCCAGGCATAGCCTCATTGGAATGAGAAAAACCTTAGTGTTTTACAAAGGAAGAGCTCCAAATGGACAAAAGTCAGATTGGATCATGCATGAATACCGTCTTGAAACAAATGAAAATGGAACTCCTCAGGAAGAAGGATGGGTGGTGTGTAGGGTGTTCAAGAAACGAATGCCCACAATGCGGAAAGTGGGTGACTACGAGTCACCATGTTGGTACGATGACCAAGTCTCCTTCTTGCCAGAAATAGATTCTCCAAGGCGAATTCCTCAGCCTTATACATCCTACCATCTCCACTATCCATGCAAACAAGAGCTTGAGTTGCAGTATAATATCAATATGCCTCACCTTCATGGCGACCCTTTCCTCCAGCTTCCTCAGTTAGAAAGCCCCAAAGTTCCACAATCAGCAGCCAGCGTAAGTTGCAACTCAGCAATTCCATATGGTTCATATGATAGAAACAATGGGAGTACTTTGCAGTCCTCGACGCTCACACAAGAAGATCAACACACGCAACAAGGCAATACTCAACAGGATTTGCACTCACTTTACAGTAACAACAATGAGCAAGCTGTGGATCAAGTGACAGATTGGAGAGTCCTTGACAAATTTGTGGCATCTCAGCTAAGCCATGAGGATGCTTCCAAGGAAACCAACTATTCCAATGCATCAGTCTTTCAAGTGGCCGAACAAATTAATATACTAGCCAATGCATCCAAAAGGCCTTTAGAGATTGCTCAAGAGTATGCCTCAACATCTACCTCGAGTTGCCAAATAGACCTGTGGAAA

**>Unigene0016058** 257 PREDICTED: probable WRKY transcription factor 41 [Phoenix dactylifera]

GAAGGATCCCTTGACGATGGCTATAAATGGAGAAAATATGGACAGAAAGACATCCTGGGAGCCAATCATCCAAGGGGCTATTATAGGTGCATACACAGACATTCCCAGGGCTGTTTGGCTACAAAGCAAATGCAAAGAACAGAAGAAGATCCACTTATCTTTGATGTCACGTACAGAGGAACACACACTTGTCAGATGGACACCATCAGAGACCCGCAGGGTTTGGAACCTAACGTCCCTGACTTA
